# Dysregulation of RNA interference components in COVID-19 patients

**DOI:** 10.1186/s13104-021-05816-0

**Published:** 2021-10-29

**Authors:** Seyyed Reza Mousavi, Maryam Sadat Sajjadi, Farinaz Khosravian, Sara Feizbakhshan, Sharareh Salmanizadeh, Zahra Taherian Esfahani, Faeze Ahmadi Beni, Ameneh Arab, Mohammad Kazemi, Kiana Shahzamani, Ramin Sami, Majid Hosseinzadeh, Mansoor Salehi, Hajie Lotfi

**Affiliations:** 1 Cellular, Molecular and Genetics Research Center, Isfahan University of Medical Sciences, 8175954319 Isfahan, Iran; 2 Medical Genetics Research Center of Genome, Isfahan University of Medical Sciences, Isfahan, Iran; 3 Medical Genetics Laboratory, Alzahra University Hospital, Isfahan University of Medical Sciences, Isfahan, Iran; 4 Department of Genetics and Molecular Biology, School of Medicine, Isfahan University of Medical Sciences, Isfahan, Iran; 5 Noor Educational and Medical Center،Isfahan University of Medical Sciences, Isfahan, Iran; 6 Isfahan Gastroenterology and Hepatology Research Center (lGHRC), Isfahan University of Medical Sciences, Isfahan, Iran; 7 Department of Pulmonology, School of Medicine, Isfahan University of Medical Sciences, Isfahan, Iran; 8 Craniofacial and Cleft Research Center, Isfahan University of Medical Sciences, Isfahan, Iran; 9 Department of Medical Biotechnology, Faculty of Advanced Medical Sciences, Tabriz University of Medical Sciences, Tabriz, Iran

**Keywords:** SARS-CoV-2, RNA interference, Dicer, Drosha, Ago2, DGCR8

## Abstract

**Objective:**

Severe acute respiratory syndrome coronavirus 2 (SARS-CoV-2) is the novel coronavirus causing severe respiratory illness (COVID-19). This virus was initially identified in Wuhan city, a populated area of the Hubei province in China, and still remains one of the major global health challenges. RNA interference (RNAi) is a mechanism of post-transcriptional gene silencing that plays a crucial role in innate viral defense mechanisms by inhibiting the virus replication as well as expression of various viral proteins. Dicer, Drosha, Ago2, and DGCR8 are essential components of the RNAi system, which is supposed to be dysregulated in COVID-19 patients. This study aimed to assess the expression level of the mentioned mRNAs in COVID-19patients compared to healthy individuals.

**Results:**

Our findings demonstrated that the expression of Dicer, Drosha, and Ago2 was statistically altered in COVID-19 patients compared to healthy subjects. Ultimately, the RNA interference mechanism as a crucial antiviral defense system was suggested to be dysregulated in COVID-19 patients.

**Supplementary Information:**

The online version contains supplementary material available at 10.1186/s13104-021-05816-0.

## Introduction

Severe acute respiratory syndrome coronavirus 2 (SARS-CoV-2) is a novel coronavirus responsible for severe respiratory disease (COVID-19). The virus was first identified in Wuhan, Hubei, China, in 2019 and spread worldwide. After the Chinese epidemic peak, other countries, especially South Korea, Italy, and Iran saw a significant spread of the disease. [1–3]. SARS‐CoV‐2 categorized in beta-coronaviruses lineage and is closely related to the SARS‐CoV virus [4, 5]. The respiratory system and lungs are most susceptible to damages resulting from the virus that leads to failed functions of the respiratory system, severe acute pulmonary disorders, and consequent mortality [6, 7]. Coronavirus is capable of producing double-stranded RNA (dsRNA) during the infection cycles. Host cell pattern recognition receptors (PRRs) could distinguish viral dsRNA as a foreign pathogen and respond to them by activating several antiviral pathways such as the RNA interference (RNAi) mechanism that is critical for early defense against viral invasion [8]. RNAi is a mechanism of post-transcriptional gene silencing in eukaryote and human cells, that inhibit virus replication and expression of various viral proteins. The RNAi system as an innate immunity pathway is mainly mediated by two non-coding molecules, including microRNA (miRNA) and small interfering RNA (siRNA) [9]. miRNA biogenesis involves Drosha, Dicer and RNAase III proteins, while siRNA processing is exclusively associated with Dicer function. Initially, endogenous or exogenous dsRNAs bounds to form siRNA or miRNA-induced silencing complex (RISC). The most critical component of these complexes is argonaute RISC catalytic component (AGO) proteins, Ago2 in particular. Using a guide RNA strand, the RISC complex could eighter suppress the mRNA translation or degrade the mRNA by Ago slicer activity [10]. Identification of exogenous dsRNAs through PRRs results in the generation of siRNAs (21—25nt in length) mediated by Dicer. One of the siRNA strands bound the RISC complex RISC and cleaves the target mRNA [11]. Similarly, during viral infection, the dsRNA of the viruse is cleaved by Dicer to generate siRNAs, which finally target the complementary viral RNA [12]. The RNAi mechanism could inhibit different types of virus infections such as rotavirus [13], influenza virus [14], and HIV-1[15, 16]. Moreover, siRNA duplexes could effectively reduce the expression of SARS-CoV genes [9]. In addition, viruses are able to encode specific suppressive proteins, which could disrupt the RNAi-mediated host defense. Thus, the balance between host RNAi components and RNAi viral inhibitors could determine disease outcomes [17]. In spite of recent advances in the development of vaccines against SARS-CoV-2, it is still remaining one of the major healthcare challenges worldwide. Thereby, providing detailed insights into the molecular pathogenesis mechanism is highlighted. Dicer, Ago2, DGRC8, and Drosha are the four critical components of RNAi system involved in the innate antiviral defense. To the best of our knowledge, no study has been reported on the RNAi mechanism dysregulation in COVID-19 patients. Therefore, here the expression levels of these genes were evaluated to predict the probable dysregulation of this pathway resulted from COVID-19**.**

## Main text

### Materials and methods

#### In silico* analysis: Gene Ontology protein–protein interaction (PPI) network analysis*

To find the probable molecular roles of the selected genes, gene ontology analysis was carried out by Cytoscape ClueGO plug-in [18]. Further, to predict the correlation and interaction of genes expression levels, PPI network was depicted through string database [19] and Cytoscape string plug-in [20].

#### Sampling

A case group consisted of 20 COVID-19 patients and 20 healthy individuals recruited from Alzahra Hospital (Isfahan, Iran). Patients were hospitalized in intensive care unit (ICU) with severe symptoms and their status were diagnosed by an infectious specialist. The selected patients had severe symptoms with no history of an underlying disease and autoimmune disorders. The characteristics of patients are mentioned in Additional file 1: Table S1.

#### RNA extraction

Total RNA was extracted from whole blood using RNA Extraction-Kit (Favor-Prep, Blood/Cultured Cell Total RNA. Taiwan). The quality of RNA was measured at 260/280 nm by a NanoDrop spectrometer (Thermo Scientific, Waltham, MA, USA).

#### Complementary DNA (cDNA) synthesis

According to the manufacturer's protocol, cDNA synthesis was performed using a BioFACT RT-Kit (Biofact, Korea) and stored at − 20 °C until the next step.

#### Quantitative real-time PCR (qPCR)

Real-time PCR were performed using SYBR Green master mix (Biofact. Korea) and specific primers conducted on Rotor-Gene 6000 instrument (Corbett Life Science, Mortlake, Australia) as follow: 95 °C for 15 min followed by 40 cycles of 95 °C for 20 s, 60 °C for 30 s and 72 °C for 30 s. The sequences of primers are listed in Additional file 1: Table S2. The data was analyzed using the 2^−ΔΔCT^ method. The glyceraldehyde-3-phosphate dehydrogenase (GAPDH) used as internal references gene.

#### Statistical analysis

To analyze the expression levels of genes between two groups, the unpaired parametric t-test and the nonparametric Mann–Whitney test were used. All results are presented as mean ± SD (standard deviation), P ≤ 0.05 was considered statistically significant, and analysis was done by GraphPad Prism version 8.0.2 (Graph Pad, San Diego, CA, USA).

### Results

#### In silico analysis: gene ontology and PPI network analysis

**Fig. 1:**
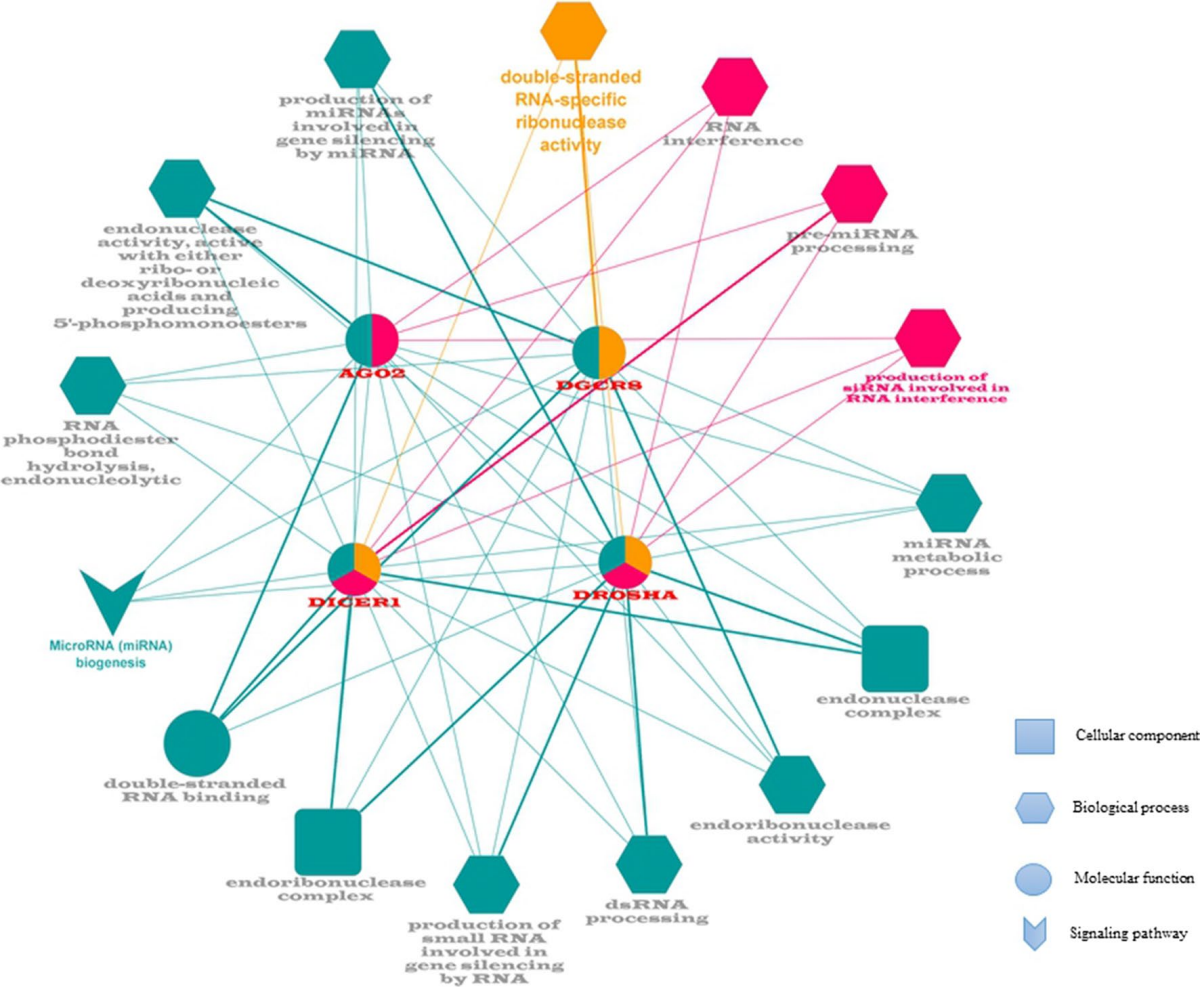
Gene ontology enrichment analysis. microRNA biogenesis was enriched as a signaling pathway in which all four genes are involved. dsRNA binding and endoribonuclease complex were enriched as molecular function and cellular compartment, respectively (p-value ≤ 0.05)

**Fig. 2:**
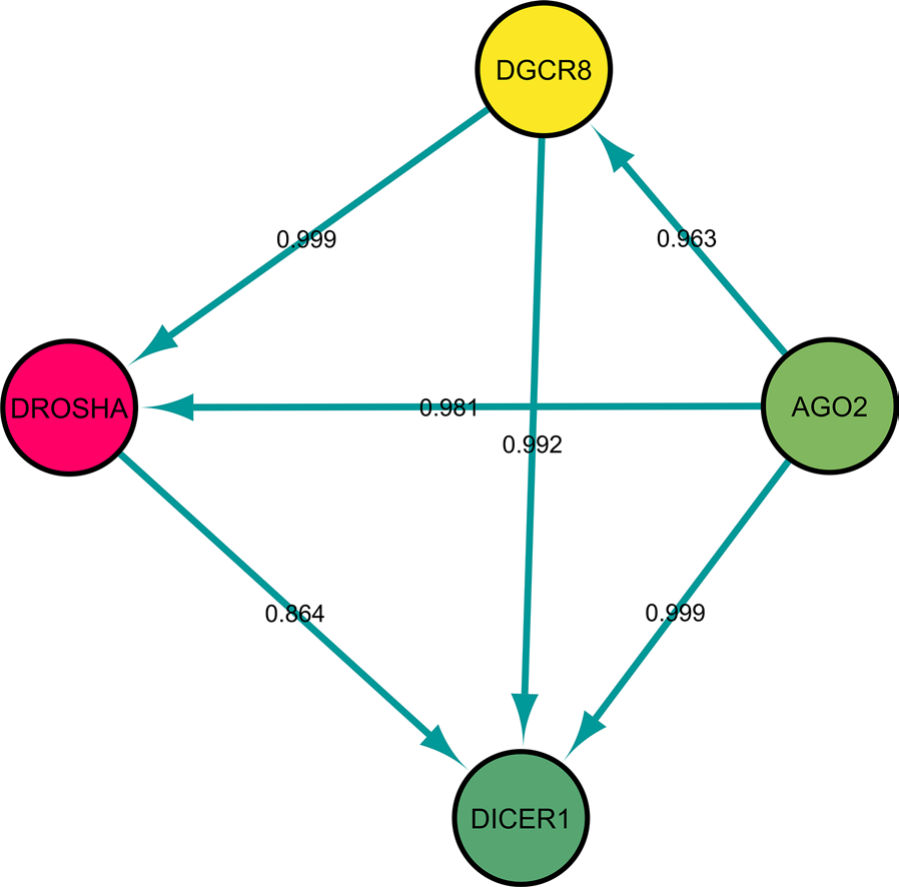
Protein–protein interaction (PPI) network analysis of DICER, Drosha, Ago2, and DGCR8. The edge's labels are based on predicted PPI score

Analyzing gene ontology and signaling pathways indicated that the Dicer, Ago2, Drosha, DGCR8 are involved in RNAi, miRNA biogenesis, and double-strand RNA binding mechanisms (Fig. 1). Moreover, using the string plugin, the PPI network determined high interrelations among proteins. (Fig. 2).

#### Expression levels of Dicer, Drosha, Ago2, and DGCR8

**Fig. 3:**
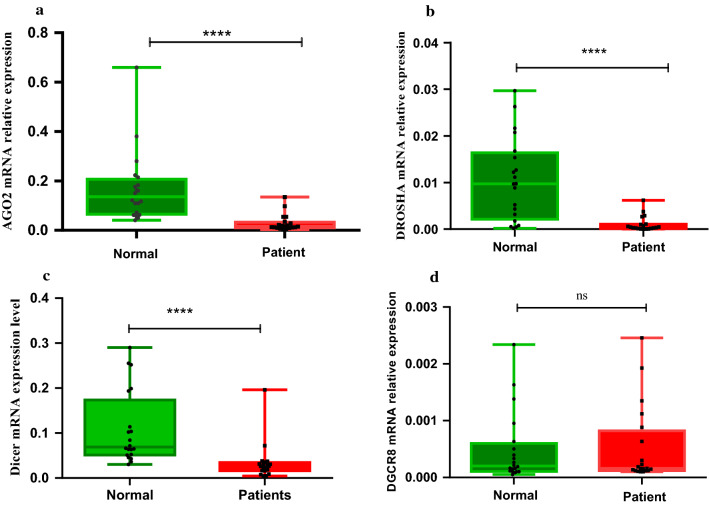
Quantitative real-time PCR analysis of Ago2 (**A**), Dicer (**B**), Drosha (**C**), and DGCR8 (**D**) in COVID-19 patients and healthy controls. The mRNA expression level of genes was evaluated in the whole blood samples of 20 COVID-19 patients and 20 controls. P values are calculated by parametric t-test for Ago2 and Dicer (p-value < 0.0001) and non-parametric Mann Whitney test for Drosha p-value < 0.0001 andDGCR8 non-significant (ns: p-value 0.213). GAPDH was utilized for an endogenous reference to normalize mRNA expression levels

Ago2 and Dicer data were normally distributed; therefore, analyzed by the unpaired parametric t-test. Conversely, Drosha and DGCR8 data were not normally distributed, thus, the non-parametric Mann–Whitney test was applied. It was determined that expression level of Ago2, Dicer*,* and Drosha were significantly downregulated (Log FC: − 3.46, − 1.92, − 4.23, respectively) in COVID-19 patients compared with control group (Fig. 3 A–C). However, no significant difference of DGCR8 expression (Log FC: − 0.32) was found groups (Fig. 3D). Finally, pearson correlation analysis determined significant interrelation between Ago2 and Drosha expression with Dicer expression level (Additional file 1: Figure S1).

## Discussion

Since the onset of the Severe Acute Respiratory Syndrome (SARS) in 2002–2003 and the Middle East Respiratory Syndrome (MERS) in 2012, many research has been devoted to providing accurate information on the pathogenesis of the disease along with proposing new treatment strategies. Despite outstanding progress in producing effective vaccines, SARS-CoV-2 remains a global health challenge throughout the world. To date, several diagnostic strategies and treatment methods have been investigated, including recovery plasma and serum therapy [21], autophagy and virophagy [22], antiviral drugs [23–26], and nanobiotechnology-based approaches. Due to the necessity developing effective therapeutics based on the virus-pathogenesis mechanism, the RNAi mechanism has the point of intense interest. Several proteins such as host receptors as well as proteins associated with the virus entrance, replication, and survival could be the potential targets for RNAi mechanism [6]. This study investigated the role of the RNAi system as an innate immune response to viral infection in COVID-19 patients. Dicer, Drosha, Ago2, and DGCR8 (critical components of RNAi) were selected for evaluation in COVID-19 patients. Based on the in-silico analysis, these genes are significantly involved in gene silencing processes through miRNA and siRNA (Fig. 1). RNAi is defined as an antiviral defense mechanism that suppresses the expression of viral proteins by targeting mRNAs [10, 27]. Hasan et.al investigated active RNAi systems against SARS-CoV-2 using computational prediction approaches. They reported 24 pre-miRNA hairpins, 131 siRNAs, 12 human miRNA, and 10 siRNA molecules as active therapeutic agents against SARS-CoV-2 [28]. Previous studies have examined the antiviral defense potential of several miRNAs and siRNAs against some viruses including HIV-1 [29], Influenza [30], Zika [31] and Hepatitis C (HCV) [32]. These information introduced some antiviral RNAi therapeutics that could effectively inactivate SARS-CoV-2 [6]. Until a few years ago, siRNAs were thought to be exclusively exogenous, like viral RNA. However, investigations have provided some strong evidence that mammalian cells could produce siRNAs against viral infection. Significantly, the production of endogenous siRNAs in 293 human cells was reported during the response to infection with influenza A virus (IAV) or human enterovirus 71 (HEV71) [33, 34]. Contrary to the antiviral function of RNAi, viruses could be released virus-encoded suppressors of RNAi (VSRs) to escape from the immunity system Some of these suppressors have been identified in cells infected with influenza A virus (non-structural protein 1), human enterovirus 71 (3A protein), and Nodamura virus (B2 protein) [35]. In addition, He et al. found that RNAi (siRNA-RISC) targets replicase 1A region of SARS-CoV, thus inhibiting SARS-CoV infection *in-vitro* [36]. Given the 80% sequence identity between SARS-CoV and SARS-CoV-2 and high protein homology [37], investigation of SARS-CoV-2 could be effective. We found that the expression levels of Dicer, Drosha, and Ago2 were significantly reduced in patients with COVID-19 compared with healthy individuals. However, the differentially expressed DGCR8 between two groups was not statistically significant. Matskevich et al. investigated the role of RNAi in both Vero and A549 cell lines lacking interferon (IFN) system that were infected with influenza A virus. They concluded that the disfunction of Dicer could increase viral replication and induce apoptosis in infected cells [38]. Moreover, Modai et al. determined that HIV-1 infection could up-regulate the miRNAs and down regulate the level of Dicer1, HRB (HIV-1 Rev-binding protein) and HIV-EP2 (Human Immunodeficiency Virus Type I Enhancer Binding Protein 2), in human Sup-T1 cells. These miRNAs could regulate directly the host genes (Dice1, HRB and HIv-EP2) and HIV-1 interferes with the human immune response against HIV infection [39]. In another study, the significant reduction of Dicer, Drosha, and DGCR8 levels were found in human A-549 cells following Dengue virus (DENV4) infection [40].

There are no reports of Ago2 expression in human cells. However, defective fly Ago-2, in *Drosophila melanogaster*, is susceptible to infection with Drosophila C virus and cricket paralysis virus [41]. Demirci et al. are predicted that about 30 viral mature miRNA-like sequences could target 1,367 human genes, affecting their functions at different cellular processes. Furthermore, some human miRNAs could target SARS-CoV-2 genes such as S, M, N, E proteins and open reading frames. Ultimately, these valuable insights on miRNAs mechanism could promise further advances in developing novel therapeutic agents for SARS-CoV-2 infections [42]. A recent in silico targeted SARS and COVID-19 genomes using human miRNAs. SARS and COVID-19 targeting miRNAs, 848 and 873 respectively, were found in which 315 and 290 miRNAs are exclusive for COVID-19 and SARS, respectively. The results also identified 19 miRNA targeting isolates out of 29 COVID-19 isolates [43]. In addition, Arisan et al. suggested seven key miRNAs linked with viral pathogenicity and host responses that could be used as effective therapeutic approaches in the treatment of COVID-19 and its pathological consequences [44]. Overall, dysregulation of Dicer, Drosha, Ago2 could play an important role in promoting viral infection. An explanation for the reported dysregulation in the three mRNAs mentioned is that SARS-COV-2 may defeat key components of the RNAi system to escape antiviral defense resulting in uncontrolled replication and transcription. On the other hand, given the high average age of patients admitted to the ICU and the clinical manifestations of the disease, it could be suggested that the RNAi mechanism is unable to defend against viral infection and the virus rapidly disables the system. Finally, correlation analysis demonstrated a significant relation between Ago2 expression to Dicer and Drosha to Dicer (Additional file 1: Figure S1). These significant interactions may indicate the interrelation of RNAi components, in which dysregulation of one factor could exacerbate the effects of the virus infection and consequently lead to the malfunction of the RNAi mechanism. As predicted in Fig. 2, these proteins may be strongly correlated based on high scores. However, according to the results of correlation analysis, only two interrelations (Ago2 with Dicer, and Drosha with Dicer) were confirmed.

## Conclusion

No vaccine or drug has been developed against COVID-19 with 100 efficiencies. In this regard, development of novel therapeutic agents considering the role of RNAi mechanism is of great importance. In conclusion, we observed the dysregulation of key RNAi components, Dicer, Ago2, and Drosha, as crucial antiviral defense factors in RNAi system in COVID-19 patients. This finding could provide insights to further identification of SARS-CoV-2 pathogenesis for COVID-19 treatment based on the RNAi system.

## Limitations

Difficulty of sampling and small size of population.

## Supplementary Information

**Additional file 1: Table S1**. Sex and age of the enrolled patients in this study. **Table S2**. Real-time primer sequences. **Figure S1**. Correlation analysis graphs. The interrelation of (A) Ago2 with Dicer (p-value ≤0.002; r: 0.7430; 95% confidence interval; 0.4477 to 0.8922; R squared: 0.5521) and (B) Drosha with Dicer (p-value ≤ 0.0034; r: 0.6223; 95% confidence interval: 0.2481 to 0.8349; R squared: 0.3872) was determined with the regression line. The expression values were calculated as -log relative expression.

## Data Availability

The data that support the findings of this study are available on request from the corresponding author. The data are not publicly available due to privacy or ethical restrictions.

## Abbreviations

DENV4: Dengue virus
dsRNA: Double-stranded RNA
HEV71: Human enterovirus 71
IAV: Influenza A virus
MERS: Middle East respiratory syndrome
miRNA: MicroRNA
PPI: Protein–protein interaction
PRRs: Pattern recognition receptors
RISC: RNA-induced silencing complex
RNAi: RNA interference
SARS: Severe acute respiratory syndrome
SARS-CoV-2: Severe acute respiratory syndrome coronavirus 2
siRNA: Small interfering RNA
VSRs: Virus-encoded suppressors of RNAi
