# Supplementary material for: Dysregulation of RNA interference components in COVID-19 patients
Source: BMC Res Notes. 2021 Oct 29;14:401. doi: 10.1186/s13104-021-05816-0 (PMC8554738; doi:10.1186/s13104-021-05816-0)
Supplement: Supplementary file 1 — Additional file 1: Table S1. Sex and age of the enrolled patients in this study. Table S2. Real-time primer sequences. Figure S1. Correlation analysis graphs. The interrelation of (A) Ago2 with Dicer (p-value ≤0.002; r: 0.7430; 95% confidence interval; 0.4477 to 0.8922; R squared: 0.5521) and (B) Drosha with Dicer (p-value ≤ 0.0034; r: 0.6223; 95% confidence interval: 0.2481 to 0.8349; R squared: 0.3872) was determined with the regression line. The expression values were calculated as -log relative expression. [file 13104_2021_5816_MOESM1_ESM.docx]

| Genes | Forward primer | Reverse Primer |
| --- | --- | --- |
| AGO2 | 5'-GAACATGACAGTGCTGAAGGAA-3' | 5'-CGCAGAGTGTCTTGGTGAAC-3' |
| DICER | 5'-CGAGCCTCCATTGTTGGTCC-3' | 5'-ACTGCCTTCGTTTCGTGGAA-3' |
| DROSHA | 5'-TGCTTGACACTTAGGACAGAAGG-3' | 5'-TTCCGCTTGCTGAATACTTGGT-3' |
| DGCR8 | 5'-AACTGGGGGTCTTTACTGCG-3' | 5'-TGTCCGATGTCTCCTGCTTG-3' |
| GAPDH | 5'-AAGGTGAAGGTCGGAGTCAAC-3' | 5'-GGGGTCATTGATGGCAACAA -3' |

|  | Covid-19 (n=20) | Control (n=20) | Statistical difference (p value) |
| --- | --- | --- | --- |
| Sex (male/female) | 12/8 | 14/6 | n/a* |
| Age (years)± SD* | 61.4± 8.9 | 59.3± 8.8 | 0.4659 |

**Table S1.**Sex and age of the enrolled patients in this study

* SD: Standard deviation; n/a: not applicable

**Table S2.** Real-time primer sequences


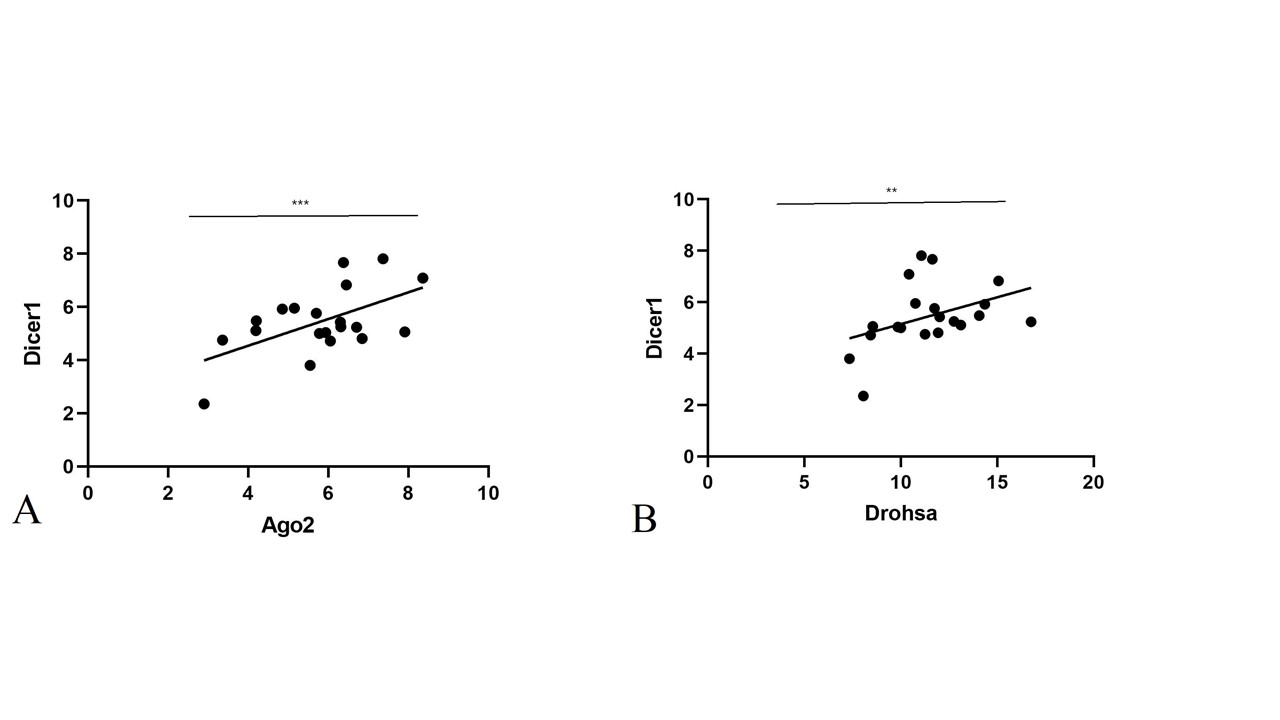


**Figure S1.**Correlation analysis graphs. The interrelation of **(A)** Ago2 with Dicer (p-value ≤0.002; r: 0.7430; 95% confidence interval; 0.4477 to 0.8922; R squared: 0.5521) and **(B)** Drosha with Dicer (p-value ≤ 0.0034; r: 0.6223; 95% confidence interval: 0.2481 to 0.8349; R squared: 0.3872) was determined with the regression line. The expression values were calculated as -log relative expression.
